# Genomic Sequence and Pathogenicity of the Chicken Anemia Virus Isolated From Chicken in Yunnan Province, China

**DOI:** 10.3389/fvets.2022.860134

**Published:** 2022-05-18

**Authors:** Manman Dai, Yuwen Huang, Lin Wang, Jing Luo, Nan Yan, Lin Zhang, Haoqi Huang, Jie Zhou, Ziwei Li, Chenggang Xu

**Affiliations:** National and Regional Joint Engineering Laboratory for Medicament of Zoonosis Prevention and Control, Guangdong Provincial Key Laboratory of Zoonosis Prevention and Control, College of Veterinary Medicine, South China Agricultural University, Guangzhou, China

**Keywords:** chicken anemia virus, virus isolation, high pathogenicity, immunosuppression, pathogenicity analysis

## Abstract

Chicken anemia virus (CAV), which has been reported in many countries, causes severe anemia and immunosuppression in chickens. In this study, a CAV strain YN04 belonging to genotype A was first identified from infected chickens in Yunnan province, China. Moreover, the animal infection experiments further confirmed that the strain YN04 is a highly pathogenic strain, which can cause 86.67% mortality in chickens in the infection group. The mean death time of infected chickens was 13.1 days post infection (dpi). CAV infection induced severe anemia with significant decrease in packed cell volume (PCV), and serious atrophy and lesion of thymus and bursa with high viral load at 14 dpi. Besides, CAV infection caused a sharp decrease in chicken body weight and immune organ indices including the ratio of thymus or bursa to body weight at 21 dpi, which displayed the potential immunosuppression state at this stage. These findings enrich the epidemiological data on CAV and may provide information for preventing its further spread in Yunnan province, China.

## Introduction

Chicken anemia virus (CAV) is a member of the genus *Gyrovirus*, which is a non-enveloped, icosahedral single-stranded DNA virus (1). The full length of CAV genome consists of 2,298 or 2,319 nucleotides, which contains three overlapping open reading frames (ORFs), and encodes viral protein 1 (VP1, ORF1, 52 kDa), VP2 (ORF2, 24 kDa), and VP3 (ORF3, 13 kDa) proteins respectively (2, 3). VP1 is the major viral structural protein playing a critical role in viral growth and spread, while VP2 is a scaffold protein that in combination with VP1 generates neutralizing epitopes (4, 5). Molecular characterization of the CAV genome revealed that the VP1 protein had a hypervariable region (positions 139–151) (6). It was reported that the AA at position 394 in VP1 was a major genetic determinant of virulence. Q394 (glutamic acid at 394 position) presenting in VP1 protein indicates the potentially highly pathogenic strain of CAV. Instead, H394 (histidine at 394 position) in VP1 protein implies a moderately pathogenic strain of CAV (7–10). VP3 is widely known as apoptin, as it facilitates apoptosis in the transformed cells, making it a potential agent in the control of cancer disease (11). All CAV isolates belong to one serotype, but different genotypes including genotype A, B, and C have been reported (12, 13).

Chicken infectious anemia (CIA) caused by CAV, is an economically important disease that affects poultry industry globally (14–16). Chickens of all ages are susceptible to infection, but susceptibility to anemia rapidly decreases in immunologically intact chickens during the first 1–3 weeks of life (17–19). CIA is an extremely epidemic immunosuppressive disease in young chickens, which is characterized by severe anemia, weight loss, aplasia of bone marrow, and generalized lymphoid atrophy with concomitant immunosuppression (20). CAV can spread both horizontally and vertically (21, 22). In adult birds, the virus serves as an important cofactor for establishment of other viral infections, such as Marek's Disease virus (MDV), reticuloendotheliosis virus (REV), infectious bursal disease virus (IBDV), avian leukosis virus subgroup J (ALV-J), reovirus, and adenovirus (23, 24).

Previously, CAV has been reported by many countries including Israel, Egypt, and Cambodia (25–27). Also, in China, CAV infections have been detected in Guangdong, Shandong, Beijing, Henan, Hebei, and Taiwan provinces (2, 16, 28, 29). In this study, we first reported the CAV infection in Yunnan province, China. We also investigated the genetic and pathogenic characteristics of the isolated YN04 strain. Our findings would enrich the epidemiological data on CAV and may provide information for preventing its further spread in Yunnan province, China.

## Materials and Methods

### Ethics Statement

All animal research projects were sanctioned by the South China Agricultural University Institutional Animal Care and Use Committee (Identification code: 2021f005, 1 March 2021). All animal procedures were performed according to the regulations and guidelines established by this committee and international standards for animal welfare.

### Virus Isolation

A total of 8 clinical liver samples from chickens showing the symptoms of anemia, weight loss, atrophy of thymus, and pale bone marrow were collected from one breeder farm with 5% mortality, in Yunnan province, in 2020. Total DNA and RNA were extracted individually from each clinical sample using a HiPure Viral DNA/RNA Kit (Magen, Guangzhou, China), and subjected to polymerase chain reaction (PCR) or reverse transcription PCR for the detection of potential pathogens, including CAV, ALV, MDV, REV, avian influenza viruses (AIV), Newcastle disease virus (NDV), and IBDV.

The CAV-positive samples were frozen and thawed three times, homogenized in phosphate buffer saline (PBS) containing penicillin (1,000 U/ml) and streptomycin (1,000 μg/ml), and then clarified at 1,000 r/min for 5 min. These supernatants were heated at 70°C for 5 min and then filtered through a 0.22-μm filter. MDCC-MSB1 cells cultured in RPMI-1640 medium (Gibco, Carlsbad, CA, USA) with 10% FBS (Gibco, Carlsbad, CA, USA) at 37°C with 5% CO_2_ were kept in our lab. The filtered supernatants were infected with MDCC-MSB1 in 6-well-plates for 4 days. After three consecutive passages, the infected cells were harvested, repeatedly frozen and thawed three times, and centrifuged at 440 × g for 5 min, and the supernatant was collected and stored at −80°C. The DNA extracted from the supernatant of third-generation infected cells was tested by PCR using CAV-specific primers, CAV-JC. The primers CAV-JC sets were 5′-GCGGACGGGTCTAAATCA-3′ and 5′- TCTCGCCTTGTGGTGGTT-3′ (30).

### Indirect Immunofluorescence Assay Identification

Indirect immunofluorescence assay (IFA) was also used to confirm that the virus isolated from the infected cells was CAV. MDCC-MSB1 cells were infected with 300 μl viral supernatants or PBS. At 72 h post infection, the MDCC-MSB1 cells were washed three times with PBS and fixed with cold 95% ethanol at 4°C for 30 min. A CAV-specific polyclonal antibody (LSBio, Seattle, USA) was used to detect the VP3 protein. Binding of the primary antibodies was detected using Alexa Fluor 488-conjugated goat anti-rabbit IgG (Sangon Biotech, Shanghai, China) and a fluorescent microscope (Nikon, Japan). The experiment was performed in triplicate.

### Sequencing Analysis

The titer of this CIAV isolate strain was measured by chicken embryos inoculation using the Reed–Muench method and was presented as the EID_50_/0.1 ml (31, 32). Three pairs of primers from published literature were used to obtain the genome of the CAV isolate, named YN04 strain (Genbank accession number: MZ540762) (30). All PCRs were carried out using 2 × Taq Master Mix (Vazyme, Nanjing, China). PCR products were excised from 1.0% agarose gel, purified using the Gel Extraction Kit (Omega, USA), and cloned into the TA vector pMD19-T (Takara, China). Three different clones of each fragment were confirmed by sequencing (Sangon Biotech, Shanghai, China). Sequence assembly was carried out using the SeqMan program of the DNASTAR package (DNASTAR, USA). To more precisely characterize the genetic origin of the isolated strain, a phylogenetic tree based on the sequences of new isolate and 45 reference strains available in the GenBank database was generated using the MEGA program (Version 7.0). Sequences of isolated strain YN04 were compared with those of other strains present in GenBank using the Clustal W method in the MegAlign program of the DNASTAR package (DNASTAR, USA). The detailed information of CAV strains downloaded from GenBank is listed in Supplementary Table 1.

### Pathogenicity Experiments

To determine the pathogenicity of this virus in chickens, a total of 30 one-day-old specific-pathogen-free (SPF) chickens (Guangdong Da Hua Nong Animal Health Products Co., Ltd., Guangdong, China) were randomly allocated into two groups, namely, CAV infection group and control group, each with 15 chickens per group, which were housed in separate isolators. The 1-day-old SPF chicks were intramuscularly inoculated at the dose of 0.3 ml (10^4.5^EID_50_/0.1 ml) of strain YN04. The control group was injected with 0.3 ml PBS alone. All chickens were observed for clinical symptoms of mortality until 21 days. The mean death time was calculated as [[number of dead chickens at x days post-infection (dpi)] × x + (number of dead chickens at y dpi) × y] / total number of dead chickens. The weight of each chicken in the control group and infection group was measured at 0 dpi, 7 dpi, 14 dpi, and 21 dpi. Serum samples were collected from both groups at 0 dpi, 7 dpi, 14 dpi, and 21 dpi for detecting the virus-specific antibody level using commercially available CAV antibody ELISA test Kit (IDEXX, Maine, USA). Sera samples were diluted 10-fold and tested according to the manufacturer's recommended protocol.

To determine the tissue distribution of this virus, 21 chickens were intramuscularly inoculated at the dose of 0.3 ml (10^4.5^EID_50_/0.1 ml) of strain YN04, while 12 chickens inoculated with 0.3 ml PBS was the control group. At 7 dpi, 14 dpi, and 21 dpi, 3 chickens of each group were randomly chosen and euthanized. Samples of thymus, spleen, liver, and bursa of Fabricius were excised from each chicken. After weighing the organs, the immune organ indices were calculated as organ weight (wet weight, mg) / body weight (g) ×100%. The representative tissue samples including thymus, spleen, and bursa of Fabricius were collected in 10% buffered formalin. Formalin-fixed tissues were processed routinely through graded ethanol, xylene, and paraffin embedding to obtain 5 mm thick sections, and stained with hematoxylin and eosin (H&E) stain for histopathological examination following the standard technique.

### Packed Cell Volume Detection

The packed cell volume (PCV) (hematocrit values) was assessed with micro-hematocrit capillary tube method. Blood samples were collected from both the control and infection groups at 7 dpi, 14 dpi, and 21 dpi. According to the published paper, chickens can be regarded as anemic when their PCV is <27% (33).

### Real Time-PCR Assay

Viral loads in various tissue organs were analyzed by a quantitative real-time PCR (qRT-PCR). Total DNA from samples of tissue organs including thymus, spleen, liver, and bursa of Fabricius or blood were extracted using HiPure Viral DNA Mini Kit (Magen, Guangzhou, China) or HiPure Blood DNA Mini Kit (Magen, Guangzhou, China) according to the manufacturer's instructions. qRT-PCR was performed on an ABI7500 Real-Time PCR system (Applied Biosystems, USA) using ChamQ Universal SYBR qPCR Master Mix (Vazyme, Nanjing, China). One specific CAV-VP2 primer sets used for qRT-PCR was 5′-ATGGCAAGACGAGCTCGC-3′ and 5′-TCACACTATACGTACCGGGG-3′ (23).

### Statistical Analyses

Statistical comparisons were made by GraphPad Prism 8 (GraphPad Software Inc., San Diego, CA, USA). The results were presented as mean ± standard error of the mean (SEM). The unpaired *t*-test was used for statistical comparison. The survival rate of chickens was calculated using log-rank (Mantel-Cox) tests and Gehan-Breslow-Wilcoxon tests. ns indicates not significant. ^*^p < 0.05, ^**^p < 0.01, ^***^p < 0.001.

## Results

### Virus Isolation and Identification

**Figure 1:**
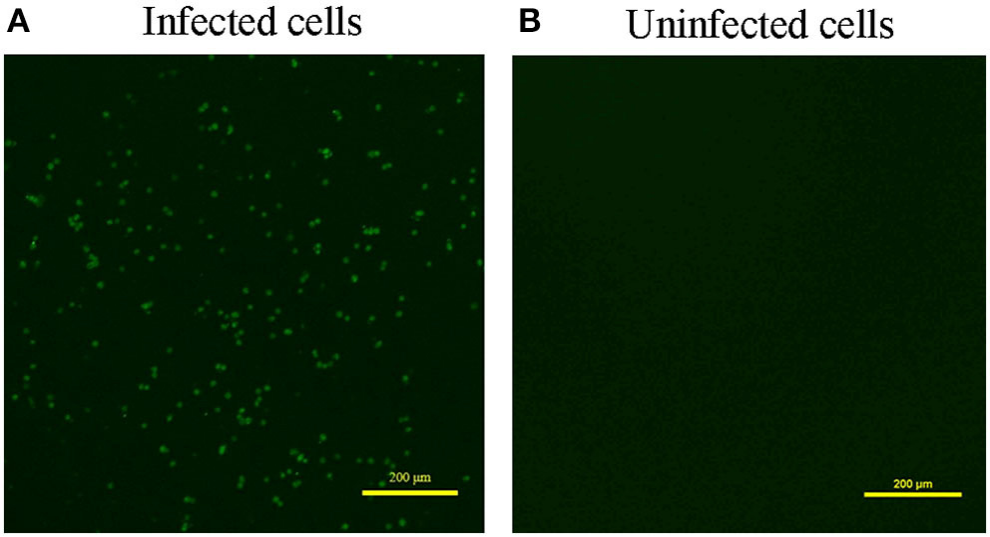
Result of Immunofluorescence assay (IFA). **(A)** Infected cells. **(B)** Uninfected cells. IFA detection of infected MDCC-MSB1 cells and uninfected MDCC-MSB1 cells. Specific staining of the infected cells with VP3 monoclonal antibody was observed by fluorescence microscopy. Scale bar: 200 μm.

Overall, 87.5% (7 out of 8) of the samples were CAV positive when defected by PCR (Supplementary Figure 1A). Meanwhile, ALV, MDV, REV, AIV, NDV, and IBDV were detected negative for all the samples with the virus-specific primers (data not shown). Besides, we used three pairs of primers to obtain the complete genome of the CAV (Supplementary Figures 1B–D), and acquired the same CAV genome sequence from the 7 CAV positive liver tissues. Next, we performed viral isolation from CAV positive liver tissues on MDCC-MSB1 cells. After three consecutive passages, the infected cells were harvested and used for identification. IFA result showed that a green fluorescent signal was observed in the infected MDCC-MSB1 cells (Figure 1A), but no signal was detected in the uninfected cells (Figure 1B). PCR and sequencing results further confirmed the successful isolation of CAV YN04 strain (Genbank accession number: MZ540762) from the third-generation infected cell culture. The virus titer of YN04 strain was detected as 10^4.5^EID_50_/0.1mL.

### Genomic Sequence

**Figure 2:**
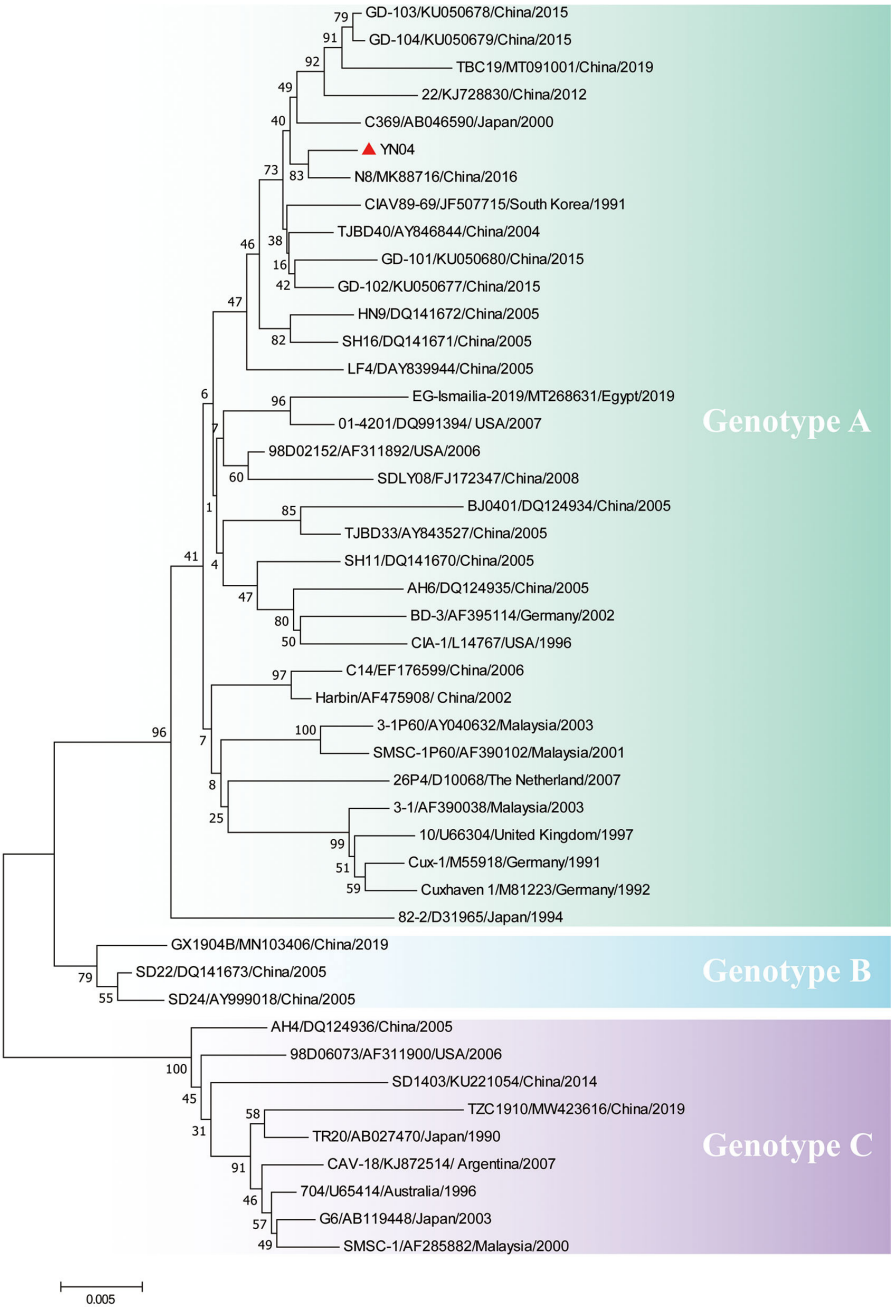
Phylogenetic analysis of YN04 based on whole genome sequence. The genomic nucleotide sequences of the YN04 strain, along with 45 chicken anemia virus (CAV) genome sequences downloaded from the GenBank database, were used for phylogenic analysis by the neighbor-joining method, with phylogenetic distances calculated using MEGA 7.0 software. Bootstrap values obtained from 1,000 replicates are shown at the major nodes. The genogroups are indicated. The strains isolated in this study are indicated by solid dots.

Sequencing of the YN04 strain revealed a genome of 2,298 bp, and phylogenetic analysis based on the complete genome sequences indicated that the YN04 strain belonged to genotype A, which was mainly epidemic in Asia (Figure 2). Besides, VP1 phylogenetic analysis result also suggested that it was falling within genotype A (Supplementary Figure 2). Position 394 in VP1 protein of strain YN04 was glutamine (Q). Position 157 in VP1 protein of strain YN04 was Methionine (M) when Position 157 in VP1 protein of most CAV isolate strains was valine (V). However, no mutations were identified in the VP2 and VP3 proteins of the YN04 strain.

### Body Weights and Immune Organs Indices

**Figure 3:**
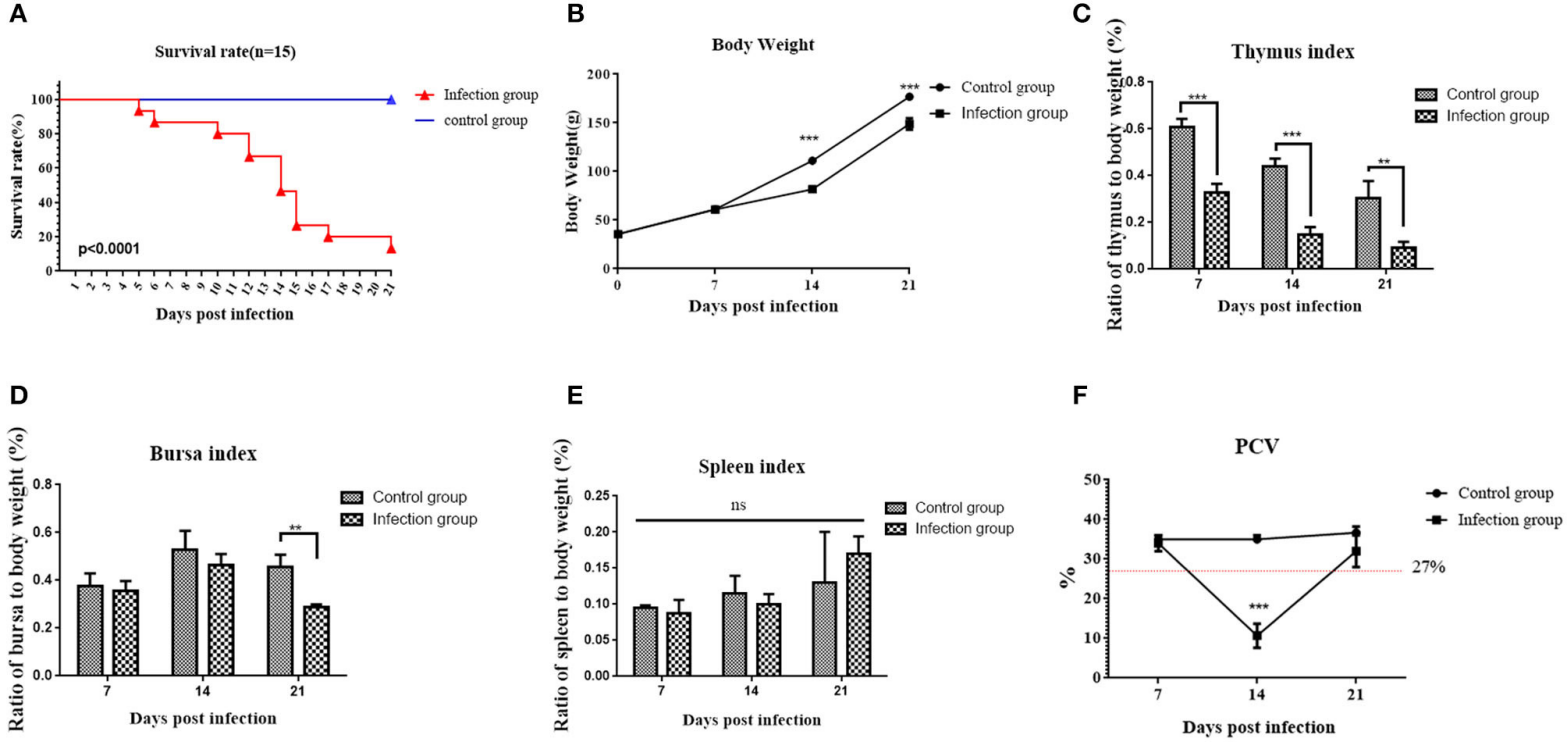
Detection of survival rate, body weights, immune organs indices, and packed cell volume (PCV) at various detected time points. **(A)** Survival rates of the control group and infection group of chicks (*n* = 15). Curves are significantly different (*p* < 0.001) by log-rank test and Gehan-Breslow-Wilcoxon analysis. **(B)** Chicken body weights in the control and infection groups. **(C)** Thymus index (the ratio of thymus to body weight) in the control and infection groups. **(D)** Bursa index (the ratio of bursa to body weight) in the control and infection groups. **(E)** Spleen index (the ratio of spleen to body weight) in the control and infection groups. **(F)** Detection of PCV (hematocrit values) at various detected time points. A PCV value below 27% was regarded as anemia. The unpaired *t*-test was used for statistical comparison. Statistical significance was assessed at *P*-values. ns *P* > 0.05, **P < 0.01, ***P < 0.001.

A total of 86.67% mortality (13 out of 15) was recorded in the CAV infection group during the experimentation (Figure 3A). Moreover, the mean death time of infected chickens was 13.1 dpi. The body weights of chickens in the infection group were significantly decreased (*p* < 0.001) at 14 dpi and 21 dpi compared to that of chickens in the control group (Figure 3B). Besides, the thymus index (the ratio of thymus to body weight) sharply decreased from 7 dpi to 21 dpi (*p* < 0.001) in the infection group (Figure 3C). Also, the bursa index (the ratio of bursa to body weight) obviously declined in the infection group at 21 dpi (*p* < 0.05) (Figure 3D). However, there was no significant change in the spleen index (the ratio of spleen to body weight) between the infection group and control group (Figure 3E).

### Hematological Changes

The PCV was measured using heparinized blood collected from chickens at 7 dpi, 14 dpi, and 21 dpi. Compared to the control group, the PCV of chickens in the infection group showed a significant decrease (*p* < 0.05) at 14 dpi, even lower than 27%, which indicated anemia in chickens of the infection group (Figure 3F).

### Gross Lesions and Histopathological Changes

**Figure 4:**
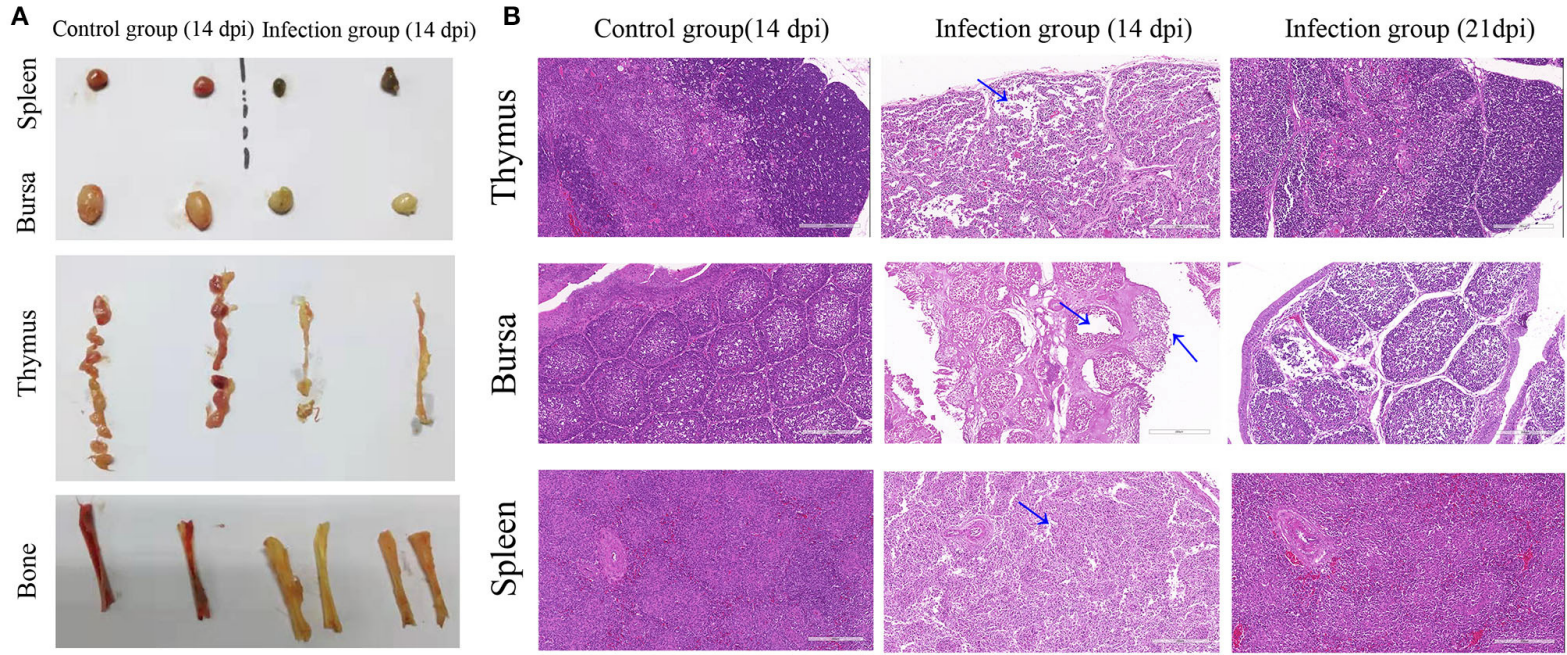
Gross lesions and histopathological changes. **(A)** Representative images of the macroscopic appearance of the spleen, bursa, thymus, and bone in the control group and infection group. **(B)** Thymus, bursa, and spleen tissues from an uninfected chicken (14 dpi) and CAV-infected chickens (14 dpi and 21 dpi). Histopathological changes are indicated by blue arrows. Hematoxylin and eosin (HE) staining, scale bar: 200 μm.

In CAV-infected chickens, severe atrophy of thymus and bursa of Fabricius, mild to moderate atrophy of spleen, and pale bone marrow were observed (Figure 4A). Histopathological analysis of organ tissues showed that characteristic tissue lesions appeared at 14 dpi and gradually returned to normal at 21 dpi. Histopathology of the spleen indicated that the number of lymphocytes decreased at 14 dpi. Histopathology of the thymus indicated that the structure of thymus lobules was vague. Many thymocytes of thymus were missing in the thymus lobule at 14 dpi. Histopathology of the bursa indicated that mucosal epithelial cells were necrotic and exfoliated at 14 dpi (Figure 4B).

### Viral Loads in Various Immune Tissues

**Figure 5:**
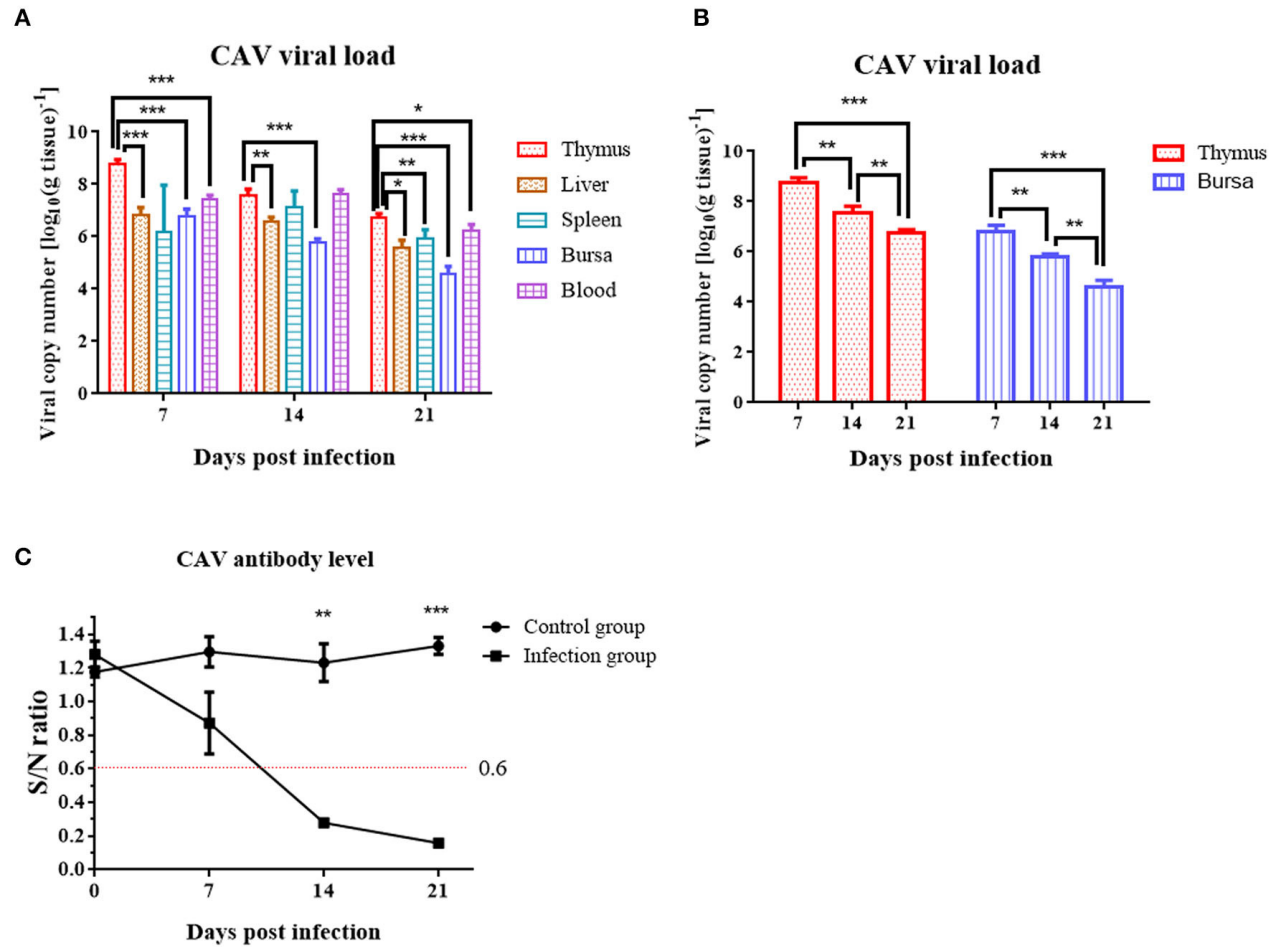
Viral loads in various organs and dynamic detection of the CAV-specific antibody level at 7 dpi, 14 dpi, and 21 dpi. **(A)** Viral loads in various organs. The copy number of the thymus compared to blood, liver, bursa, and spleen at 7 dpi, 14 dpi, and 21 dpi. **(B)** Viral loads in thymus and bursa. **(C)** Dynamic detection of the CAV-specific antibody. An S/N value below 0.6 was considered CAV antibody positive. The unpaired *t*-test was used for statistical comparison. *p < 0.05, **p < 0.01, ***p < 0.001.

At 7 dpi, 14 dpi, and 21 dpi, samples of the liver, thymus, spleen, bursa of Fabricius, and blood were excised for DNA extraction from 3 chickens randomly chosen from the infection group, to measure the CAV viral loads in various tissues. CAV was positive in each detected tissue organ from 7 dpi to 21 dpi (Figure 5A). The highest virus copy number was found in thymus (log_10_ 8.76 ± 0.18) compared to other tissues (Figure 5A, Supplementary Table 2). A general downward trend of the viral copy number in thymus and bursa was observed from 7 dpi to 21 dpi (Figure 5B).

### CAV Antibody Levels in Serum

Chicken anemia virus-specific antibodies were monitored from 0 to 21 dpi. In the control group, the CAV antibody level was all negative throughout the experiment. In the infection group, the CAV antibody level was 100% negative at 7 dpi, 100% positive at 14 dpi or 21 dpi, and displayed an upward trend from 0 to 21 dpi (Figure 5C).

## Discussion

After its first report in 1979, in Japan (34), CAV has been detected worldwide. The diversity of CAV strains in different countries has been reported previously (35–37). In recent years, CAV infections have been detected in Guangdong, Shandong, and Heilongjiang provinces of China (2, 16, 28, 38, 39). CAV was reported to attack erythroblastoid cells and thymocytes, and could cause severe anemia and immunosuppression (13). In this study, we first identified a highly pathogenic CAV strain YN04 from infected chickens in Yunnan province, China.

Here, we successfully isolated a CAV strain YN04 from CAV-positive specimens collected from one breeder farm in Yunnan province. To analyze the genetic variation of this strain and track its possible origin, we sequenced its whole genome. Phylogenetic analysis results showed that the YN04 strain belonged to genotype A, which was mainly epidemic in Asia. It was reported that Q394 (glutamic acid at 394 position) presenting in VP1 protein indicates the highly pathogenic strain of CAV. Instead, H394 (histidine at 394 position) in VP1 protein is present in a moderately pathogenic strain of CAV (7–10). Remarkably, YN04 strain had Q394 AA in VP1 protein, indicating that it is a highly pathogenic strain.

It was reported that most CAV isolate strains usually caused around 30% mortality (2, 6). Conversely, the YN04 strain isolated in this study could cause 86.67% mortality in the infection group with almost the same animal infection model, which indicated that the YN04 strain was a highly pathogenic strain. In the infected chickens, characteristic clinical signs such as weakness, anemia, and stunted growth were observed. All these changes were characteristic to CIA and were in agreement with the earlier reports (40). The obvious decrease of body weight at 14 and 21 dpi, thymus index from 7 to 21 dpi, and bursa index at 21 dpi was observed (41). Furthermore, marked changes were also observed in the hematological parameters and histopathological changes at 14 dpi. Specifically, there was a significant decrease in the PCV of infected chickens at 14 dpi (22). Severe atrophy of thymus and bursa, mild to moderate atrophy of spleen, and pale bone marrow were observed in infected chickens at 14 dpi. Besides, CAV was positive in each detected tissue organ from 7 to 21 dpi. Moreover, our study found that the viral load in thymus was highest in the infection group compared with other organ tissues (16, 22). The above results suggested that CAV is highly susceptible to infection of central immune organs including thymus and bursa.

At 14 dpi, the severe pathological damage and anemia may be related to the high viral loads in the tissue organ and blood. The tissue organs and PCV of infected chickens returned to normal at 21 dpi, which was mainly due to the increasing CAV-specific antibodies and recovery of immune organs. However, we could still detect obvious viral load at 21 dpi. It was also reported that the virus can persist in chickens even long after flock seroconversion (7, 10, 40–42). Besides, we found that the body weight, thymus, and bursa indices were significantly decreased at 21 dpi, which indicated that CAV infection may induce immunosuppression in infected chickens at this stage. The immunosuppression caused by CAV increases the susceptibility of infected chickens to other diseases such as NDV, fowl adenovirus type 4, AIV (H9N2), and infectious bronchitis virus (26, 43, 44).

In conclusion, the present study is the first report of an isolation of CAV from infected chickens in Yunnan province, with the determination of the complete genomic sequence. The strain YN04 is a highly pathogenic strain and clearly pathogenic to chickens. The strain YN04 can cause severe anemia and immunosuppression in infected chickens. This work enriches the epidemiological data and provides information for preventing its further spread in Yunnan province, China.

## Data Availability Statement

The datasets presented in this study can be found in online repositories. Sequencing data have been deposited in GenBank under the accession number MZ540762.

## Ethics Statement

The animal study was reviewed and approved by South China Agriculture University Institutional Animal Care and Use Committee (Identification code: 2021f005, 1 March 2021).

## Author Contributions

MD designed the study, performed experiments, collected and analyzed data, and revised the manuscript. YH performed experiments, collected and analyzed data, and drafted the manuscript. LW, JL, NY, LZ, HH, JZ, and ZL assisted with pathogenicity experiments and data analysis. CX coordinated the study and revised the manuscript. All authors have read and approved the final manuscript.

## Funding

This work was supported by the National Natural Science Foundation of China (32172868 and 31802174), Guangdong Basic and Applied Basic Research Foundation (2022A1515012480), Young Elite Scientists Sponsorship Program by CAST (2020QNRC001), and 111 Project (D20008).

## Conflict of Interest

The authors declare that the research was conducted in the absence of any commercial or financial relationships that could be construed as a potential conflict of interest.

## Publisher's Note

All claims expressed in this article are solely those of the authors and do not necessarily represent those of their affiliated organizations, or those of the publisher, the editors and the reviewers. Any product that may be evaluated in this article, or claim that may be made by its manufacturer, is not guaranteed or endorsed by the publisher.

## Supplementary Material

Supplementary Figure 1 Result of polymerase chain reaction (PCR). **(A)** PCR detection of chicken anemia virus (CAV) from liver samples. M, marker (DNA ladder 2000); 1~8, liver samples; NC, negative control. **(B)** PCR detection of primers CIAV-1 from CAV-positive liver tissues. M, marker (DNA ladder 2000); 1~7, CAV-positive liver tissues; NC, negative control. **(C)** PCR detection of primers CIAV-2 from CAV-positive liver tissues. M, marker (DNA ladder 2000); 1~7, CAV-positive liver tissues; NC, negative control. **(D)** PCR detection of primers CIAV-3 from CAV-positive liver tissues. M, marker (DNA ladder 2000); 1~7, CAV-positive liver tissues; NC, negative control.

Supplementary Figure 2 Phylogenetic analysis of various CAV strains based on VP1 amino acid (AA) sequences. The VP1 AA sequences of the YN04 strain, along with 45 CAV VP1 AA sequences downloaded from the GenBank database, were used for phylogenic analysis by the neighbor-joining method, with phylogenetic distances calculated using MEGA 7.0 software. Bootstrap values obtained from 1,000 replicates are shown at the major nodes. The genogroups are indicated. The strains isolated in this study are indicated by solid dots.

Supplementary Table 1 Details about the chicken anemia virus (CAV) reference strains from GenBank.

Supplementary Table 2 CAV viral load in various tissues of chickens in the infection group.
