# Supplementary figures and images for: Genomic Sequence and Pathogenicity of the Chicken Anemia Virus Isolated From Chicken in Yunnan Province, China
Source: Front Vet Sci. 2022 May 18;9:860134. doi: 10.3389/fvets.2022.860134 (PMC9158507; doi:10.3389/fvets.2022.860134)

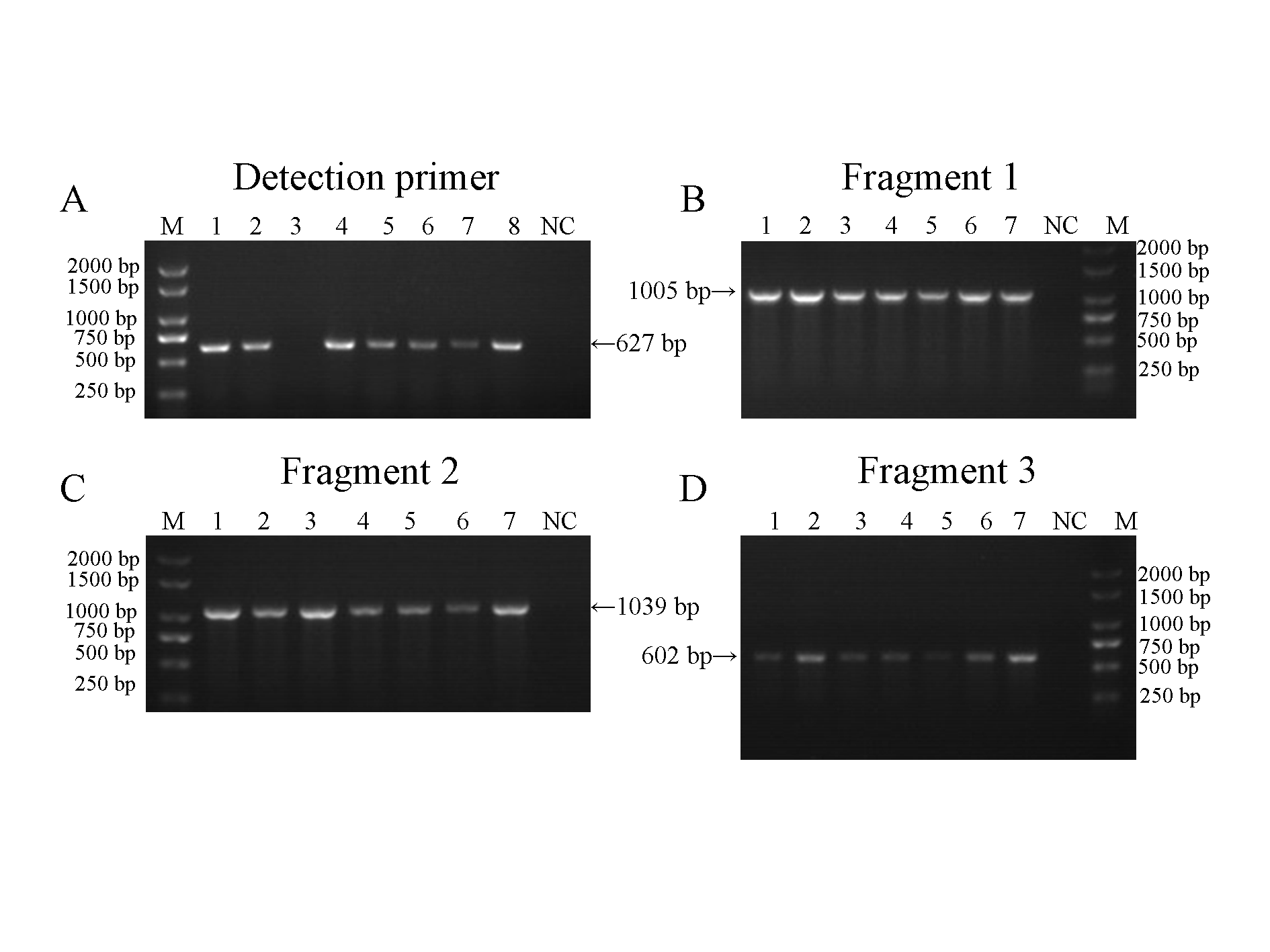

Supplement: Supplementary Figure 1 — Result of polymerase chain reaction (PCR). (A) PCR detection of chicken anemia virus (CAV) from liver samples. M, marker (DNA ladder 2000); 1~8, liver samples; NC, negative control. (B) PCR detection of primers CIAV-1 from CAV-positive liver tissues. M, marker (DNA ladder 2000); 1~7, CAV-positive liver tissues; NC, negative control. (C) PCR detection of primers CIAV-2 from CAV-positive liver tissues. M, marker (DNA ladder 2000); 1~7, CAV-positive liver tissues; NC, negative control. (D) PCR detection of primers CIAV-3 from CAV-positive liver tissues. M, marker (DNA ladder 2000); 1~7, CAV-positive liver tissues; NC, negative control. [file Image_1.TIF]

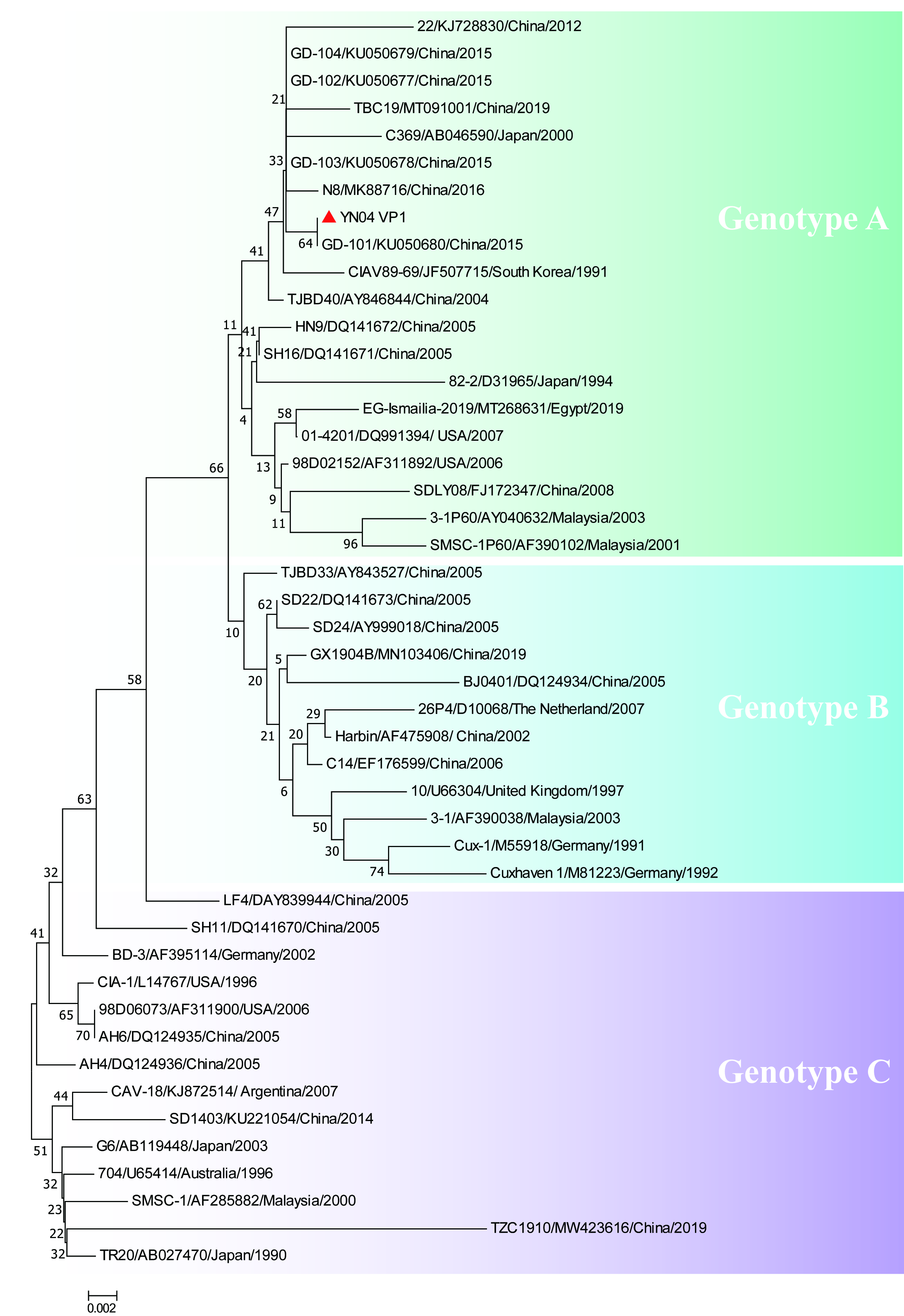

Supplement: Supplementary Figure 2 — Phylogenetic analysis of various CAV strains based on VP1 amino acid (AA) sequences. The VP1 AA sequences of the YN04 strain, along with 45 CAV VP1 AA sequences downloaded from the GenBank database, were used for phylogenic analysis by the neighbor-joining method, with phylogenetic distances calculated using MEGA 7.0 software. Bootstrap values obtained from 1,000 replicates are shown at the major nodes. The genogroups are indicated. The strains isolated in this study are indicated by solid dots. [file Image_2.TIF]
