# Supplementary material for: Genomic Sequence and Pathogenicity of the Chicken Anemia Virus Isolated From Chicken in Yunnan Province, China
Source: Front Vet Sci. 2022 May 18;9:860134. doi: 10.3389/fvets.2022.860134 (PMC9158507; doi:10.3389/fvets.2022.860134)
Supplement: Supplementary Table 1 — Details about the chicken anemia virus (CAV) reference strains from GenBank. [file Table_1.DOCX]

**Supplementary Table 1.** Details about the chicken anemia virus (CAV) strains isolated in this study and reference strains from GenBank.

| No. | Strain name | Origin | Year | Accession no. | No. | Strain name | Origin | Year | Accession no. |
| --- | --- | --- | --- | --- | --- | --- | --- | --- | --- |
| 1 | YN04 | Yunnan, China | 2020 | MZ540762 | 24 | GD-103 | Guangdong, China | 2015 | [KU050678](https://www.ncbi.nlm.nih.gov/nuccore/KU050678) |
| 2 | 3–1 | Malaysia | 2003 | [AF390038](https://www.ncbi.nlm.nih.gov/nuccore/AF390038) | 25 | GD-104 | Guangdong, China | 2015 | [KU050679](https://www.ncbi.nlm.nih.gov/nuccore/KU050679) |
| 3 | 3–1 P60 | Malaysia | 2003 | [AY040632](https://www.ncbi.nlm.nih.gov/nuccore/AY040632) | 26 | Harbin | Heilongjiang, China | 2002 | [AF475908](https://www.ncbi.nlm.nih.gov/nuccore/AF475908) |
| 4 | 10 | United Kingdom | 1997 | [U66304](https://www.ncbi.nlm.nih.gov/nuccore/U66304) | 27 | HN9 | Tianjin, China | 2005 | [DQ141672](https://www.ncbi.nlm.nih.gov/nuccore/DQ141672) |
| 5 | 98D02152 | USA | 2006 | [AF311892](https://www.ncbi.nlm.nih.gov/nuccore/AF311892) | 28 | 22 | Taiwan | 2012 | [KJ728830](https://www.ncbi.nlm.nih.gov/nuccore/KJ728830) |
| 6 | 98D06073 | USA | 2006 | [AF311900](https://www.ncbi.nlm.nih.gov/nuccore/AF311900) | 29 | LF4 | Hebei, China | 2005 | [AY839944](https://www.ncbi.nlm.nih.gov/nuccore/AY839944) |
| 7 | 704 | Australia | 1996 | [U65414](https://www.ncbi.nlm.nih.gov/nuccore/U65414) | 30 | SD22 | Shandong, China | 2005 | [DQ141673](https://www.ncbi.nlm.nih.gov/nuccore/DQ141673) |
| 8 | AH4 | Anhui, China | 2005 | [DQ124936](https://www.ncbi.nlm.nih.gov/nuccore/DQ124936) | 31 | SD24 | Shandong, China | 2005 | [AY999018](https://www.ncbi.nlm.nih.gov/nuccore/AY999018) |
| 9 | AH6 | Anhui, China | 2005 | [DQ124935](https://www.ncbi.nlm.nih.gov/nuccore/DQ124935) | 32 | SD1403 | Shandong, China | 2014 | [KU221054](https://www.ncbi.nlm.nih.gov/nuccore/KU221054) |
| 10 | BD-3 | Germany | 2002 | [AF395114](https://www.ncbi.nlm.nih.gov/nuccore/AF395114) | 33 | SDLY08 | Shandong, China | 2008 | [FJ172347](https://www.ncbi.nlm.nih.gov/nuccore/FJ172347) |
| 11 | BJ0401 | Beijing, China | 2005 | [DQ124934](https://www.ncbi.nlm.nih.gov/nuccore/DQ124934) | 34 | SH11 | Shanghai, China | 2005 | [DQ141670](https://www.ncbi.nlm.nih.gov/nuccore/DQ141670) |
| 12 | C14 | China | 2006 | [EF176599](https://www.ncbi.nlm.nih.gov/nuccore/EF176599) | 35 | SH16 | Shanghai, China | 2005 | [DQ141671](https://www.ncbi.nlm.nih.gov/nuccore/DQ141671) |
| 13 | C369 | Japan | 2000 | [AB046590](https://www.ncbi.nlm.nih.gov/nuccore/AB046590) | 36 | SMSC-1 | Malaysia | 2000 | [AF285882](https://www.ncbi.nlm.nih.gov/nuccore/AF285882) |
| 14 | 26P4 | The Netherland | 2007 | [D10068](https://www.ncbi.nlm.nih.gov/nuccore/D10068) | 37 | SMSC-1P60 | Malaysia | 2001 | [AF390102](https://www.ncbi.nlm.nih.gov/nuccore/AF390102) |
| 15 | 82–2 | Japan | 1994 | [D31965](https://www.ncbi.nlm.nih.gov/nuccore/D31965) | 38 | TJBD33 | Tianjin, China | 2005 | [AY843527](https://www.ncbi.nlm.nih.gov/nuccore/AY843527) |
| 16 | CAV-18 | Argentina | 2007 | [KJ872514](https://www.ncbi.nlm.nih.gov/nuccore/KJ872514) | 39 | TJBD40 | Tianjin, China | 2004 | [AY846844](https://www.ncbi.nlm.nih.gov/nuccore/AY846844) |
| 17 | CIA-1 | USA | 1996 | [L14767](https://www.ncbi.nlm.nih.gov/nuccore/L14767) | 40 | TR20 | Japan | 1990 | [AB027470](https://www.ncbi.nlm.nih.gov/nuccore/AB027470) |
| 18 | CIAV89-69 | South Korea | 1991 | [JF507715](https://www.ncbi.nlm.nih.gov/nuccore/JF507715) | 41 | N8 | Guangdong, China | 2016 | MK887164 |
| 19 | Cux-1 | Germany | 1991 | [M55918](https://www.ncbi.nlm.nih.gov/nuccore/M55918) | 42 | GX1904B | Guangxi, China | 2019 | MN103406 |
| 20 | Cuxhaven 1 | Germany | 1992 | [M81223](https://www.ncbi.nlm.nih.gov/nuccore/M81223) | 43 | EG-Ismailia-2019 | Egypt | 2019 | MT268631 |
| 21 | G6 | Japan | 2003 | [AB119448](https://www.ncbi.nlm.nih.gov/nuccore/AB119448) | 44 | TZC1910 | Jiangsu, China | 2019 | MW423616 |
| 22 | GD-101 | Guangdong, China | 2015 | [KU050680](https://www.ncbi.nlm.nih.gov/nuccore/KU050680) | 45 | TBC19 | Shangdong, China | 2019 | MT091001 |
| 23 | GD-102 | Guangdong, China | 2015 | [KU050677](https://www.ncbi.nlm.nih.gov/nuccore/KU050677) | 46 | 01–4201 | USA | 2007 | [DQ991394](https://www.ncbi.nlm.nih.gov/nuccore/DQ991394) |
