# Supplementary material for: Genomic Sequence and Pathogenicity of the Chicken Anemia Virus Isolated From Chicken in Yunnan Province, China
Source: Front Vet Sci. 2022 May 18;9:860134. doi: 10.3389/fvets.2022.860134 (PMC9158507; doi:10.3389/fvets.2022.860134)
Supplement: Supplementary Table 2 — CAV viral load in various tissues of chickens in the infection group. [file Table_2.DOCX]

**Supplementary Table 2.** Chicken anemia virus (CAV) viral load in various tissues of chickens in infection group.

| Organ | Days post infection | | |
| --- | --- | --- | --- |
|  | 7 | 14 | 21 |
| Thymus | 8.76 ± 0.18 | 7.56 ± 0.26 | 6.74 ± 0.13 |
| Blood | 7.41 ± 0.17 | 7.63 ± 0.12 | 6.24 ± 0.21 |
| Bursa | 6.80 ± 0.26 | 5.80 ± 0.12 | 4.60 ± 0.24 |
| Spleen | 6.16 ± 1.80 | 7.15 ± 0.59 | 5.94 ± 0.32 |
| Liver | 6.81 ± 0.30 | 6.59 ± 0.14 | 5.59 ± 0.28 |

Values are means ± SD log_10_ [DNA copy number (g tissue)^-1^].
